# Endoplasmic Reticulum Quality Control in Immune Cells

**DOI:** 10.3389/fcell.2021.740653

**Published:** 2021-09-29

**Authors:** Yalan Jiang, Zehua Tao, Hua Chen, Sheng Xia

**Affiliations:** ^1^Department of Immunology, School of Medicine, Jiangsu University, Zhenjiang, China; ^2^Department of Colorectal Surgery, Affiliated Kunshan Hospital of Jiangsu University, Kunshan, China

**Keywords:** ERQC, UPR, ERAD, autophagy, immune cell

## Abstract

The endoplasmic reticulum quality control (ERQC) system, including endoplasmic reticulum-associated degradation (ERAD), the unfolded protein response (UPR), and autophagy, presides over cellular protein secretion and maintains proteostasis in mammalian cells. As part of the immune system, a variety of proteins are synthesized and assembled correctly for the development, activation, and differentiation of immune cells, such as dendritic cells (DCs), macrophages, myeloid-derived-suppressor cells (MDSCs), B lymphocytes, T lymphocytes, and natural killer (NK) cells. In this review, we emphasize the role of the ERQC in these immune cells, and also discuss how the imbalance of ER homeostasis affects the immune response, thereby suggesting new therapeutic targets for immunotherapy.

## Introduction

The endoplasmic reticulum (ER) serves as a cell factory, playing a key role in protein synthesis, folding, and modification. However, an accumulation of misfolded or unfolded proteins in the ER leads to an ER stress response. Cells have evolved an endoplasmic reticulum quality control (ERQC) system to maintain ER homeostasis. As a complex monitoring system, the ERQC regulates ER homeostasis through three mechanisms: endoplasmic reticulum-associated degradation (ERAD), the unfolded protein response (UPR), and autophagy. ERAD and autophagy are responsible for the degradation of misfolded proteins in the ER, while constant ER stress induced by misfolded protein accumulation activates the inositol-requiring enzyme 1α (IRE1α), protein kinase RNA-like ER kinase (PERK), and activating transcription factor 6 (ATF6) signaling pathways to trigger the UPR response. Immune cells, including dendritic cells (DCs), macrophages, myeloid-derived-suppressor cells (MDSCs), B cells, T cells, and natural killer (NK) cells are key effectors in the immune system. In the development of these cells and even their activation and differentiation in the immune response, an amount of newly synthesized membrane and secretion proteins are indispensable, which first need correct intracellular folding. In this review, we summarized the recent work on the ERQC in immune cells and hope to demonstrate its role within the immune system.

## The Endoplasmic Reticulum: Structure and Function

The ER is an intracellular membranous network that is present in all eukaryotic cells. It consists of interconnected, branching membranous tubules, vesicles, and cisternae, giving rise to two main dynamic and interconvertible structures: smooth endoplasmic reticulum (SER) and rough endoplasmic reticulum (RER), with membranes of the latter being decorated by ribosomes on its outer surface (English et al., 2009; Corazzari et al., 2017). Although some cells may have little SER, all eukaryotic cells have large amounts of RER, as these play a key role in the synthesis of many integral membrane proteins and several cytosolic proteins. RER is abundant in secretory cells, such as antibody-producing plasma cells, insulin-secreting beta cells, or cells of milk-producing glands (Burgoyne and Duncan, 1998; Harding and Ron, 2002; Oyadomari et al., 2002; van Anken et al., 2009). SER, which lacks ribosomes, is not involved in protein synthesis but is responsible for the synthesis of essential lipids, such as fatty acids and phospholipids, metabolism of carbohydrates, and regulation of calcium homeostasis (Schönthal, 2012). After synthesis, proteins are translocated into the ER lumen, where they are further folded, and then dispersed to their destination. Unfortunately, these new synthesized proteins sometimes do not fold or aggregate normally (Ellgaard and Helenius, 2003). The accumulation of unfolded, misfolded, or damaged proteins leads to ER stress, which further initiates ERAD, UPR, and autophagy responses to rescue the cell from cell destruction or death.

## Endoplasmic Reticulum Stress Response and Protein Degradation

Protein folding is a complex process that depends on the interaction of chaperone proteins, folding enzymes, and glycosylases (Malhotra and Kaufman, 2007; Ron and Walter, 2007). When there is an imbalance between protein-folding capacity and protein-folding demand, this leads to an accumulation of misfolded proteins in the cell, known as ER stress (Hetz and Papa, 2018). Protein degradation is an important step in processing ER stress. The major protein degradation system includes the ubiquitin-proteasome system (UPS) and the autophagic-lysosomal system (ALS), which can selectively degrade aberrant protein fragments (Xia et al., 2020). In most cases, micromolecules and short-lived soluble proteins are retrotranslocated into the cytosol, ubiquitinated, and then degraded by the proteasomes in the ERAD system. However, when the misfolded proteins exceed ER degradation capacity, the UPR response will be activated. At the same time, aggregates of misfolded proteins that could not be processed by the proteasome are degraded through autophagy (Nam et al., 2017).

### Endoplasmic Reticulum-Associated Degradation

**FIGURE 1:**
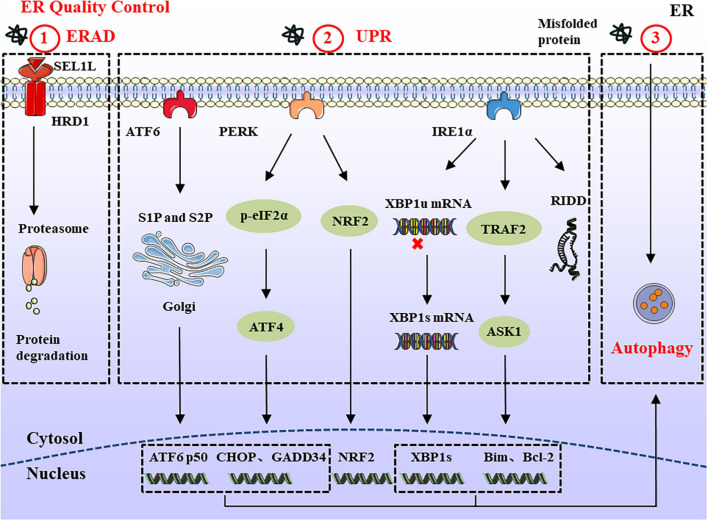
ERQC in Endoplasmic reticulum. ERQC monitors the correct folding of proteins via ERAD, UPR, and autophagy. (1) The soluble protein aggregates are retrotranslocated into cytosol through SEL1L-HRD1, where the ubiquitinated substrates are degraded by the proteasome. (2) When the misfolded proteins exceed ER degradation capacity, it leads to the ER stress response. In order to maintain ER homeostasis, the UPR response is activated which is composed of IRE1α, PERK, and ATF6 branches. The activated IRE1α catalyzes the splicing of XBP1 to generate XBP1s. In addition, IRE1α can also reduce protein synthesis through RIDD, and interacts with TRAF2 to regulate the expression of pro-/anti-apoptotic proteins. PERK reduces protein load by phosphorylating eIF2α and NRF2. Meanwhile, the upregulated ATF4 participates in cell apoptosis and protein synthesis via CHOP and GADD34. ER stress also causes the translocation of ATF6 to the Golgi apparatus, where it is spliced by S1P and S2P proteases, thereby mediating the expression of ERAD and ER chaperones. (3) In the ER stress response, large protein aggregates are degraded through the autophagy-lysosomal pathway.

Endoplasmic reticulum-associated degradation is a conserved quality control mechanism in the cell, responsible for retrotranslocation of misfolded proteins into the cytosol for proteasomal degradation (Zhou et al., 2020). The SEL1L-HRD1 protein complex comprises the most conserved members of ERAD from yeast to mammals. SEL1L is a type I transmembrane protein, which resides on the ER membrane and controls the stability of E3 ligase HRD1 (Sun et al., 2014; Jeong et al., 2016). ERAD acts in a multistep process involving recognition, extraction, polyubiquitination, and degradation (Christianson and Ye, 2014). In a simplified model of ERAD, molecular chaperones and lectins, including BiP, EDEM, ERdj, OS9, and XTP3B recognize unfolded nascent polypeptide substrates. Subsequently, unfolded proteins bind with these chaperones and are further delivered to ERAD adaptors, including SEL1L and Erlins (Qi et al., 2017). Finally, the polyubiquitinated substrates are transported to the cytosol through the retrotranslocation channel (candidates include HRD1, DER1, and DFM1) and degraded by 26S proteasome (Wu and Rapoport, 2018; Figure 1). Chronic ER stress may not necessarily trigger harmful changes, such as cell death (Hotamisligil, 2010). For example, DCs and developing B cells can tolerate ERAD deficiency and chronic ER stress, which may arise from other compensatory mechanisms, such as the UPR and autophagy (Yang et al., 2014; Ji et al., 2016). Therefore, the role of ERAD in cell development should be specifically considered according to different substrates and cell types.

### Unfolded Protein Response

When the accumulated improperly folded proteins exceed a threshold, cells activate the UPR to restore ER homeostasis. The main purpose of the UPR is to eliminate misfolded proteins and reduce the synthesis of new proteins in the ER. The UPR slows down the transcription and translation of new mRNA by degrading existing mRNA, thereby reducing the flow of new proteins into the ER lumen (Grootjans et al., 2016). The production of genes encoding molecular chaperones and foldases also increases to process the large amounts of accumulated proteins.

Mammalian cells have three ER-resident transmembrane proteins which work as sensors to detect the accumulation of unfolded or misfolded proteins: IRE1α, PERK, and ATF6 (Hetz et al., 2015; McQuiston and Diehl, 2017). Under physiological conditions, ER sensor proteins are maintained in an inactive state by interacting with glucose-regulated protein 78 (GRP78; also called BiP). As unfolded proteins accumulate, BiP dissociates from ER stress sensors and initiates the UPR. The IRE1α-X-box-binding protein 1 (XBP1) pathway is the most conserved UPR signaling branch. As a type I transmembrane protein with a cytosolic serine/threonine kinase domain, the activation of IRE1α causes conformational changes that trigger kinase activity and endoribonuclease activity to catalyze the excision of a 26-nt intron within the XBP1 mRNA, forming a stable and active transcription mRNA named XBP1s (Duwaerts et al., 2020). Furthermore, activated IRE1α forms a complex with tumor-necrosis factor-α (TNF-α)-receptor-associated factor 2 (TRAF2) at its cytosolic domain, which in turn engages apoptosis signal-regulating kinase 1 (ASK1) and JUN N-terminal kinase (JNK), leading to the activation of pro-apoptotic Bim and suppression of the anti-apoptotic activity of Bcl-2 (Schönthal, 2012; Corazzari et al., 2017). IRE1α also degrades ribosomal-associated mRNA through regulated IRE1-dependent decay (RIDD) to prevent the translation and accumulation of unfolded proteins (Coelho and Domingos, 2014). The RIDD substrate has a sequence CUGCAG similar to the stem-loop structure of XBP1 mRNA, and the activity of RIDD increased with the duration of ER stress (Maurel et al., 2014). So the basal activity of RIDD helps maintain ER homeostasis, but under prolonged ER stress, continuously increased RIDD induces apoptosis by degrading mRNAs.

The PERK arm phosphorylates eukaryotic initiation factor 2α (eIF2α) on Ser51, which reduces protein translation and lowers the protein load (Liu et al., 2015). Although the overall translation reduces after the phosphorylation of eIF2α, the expression of some key genes is upregulated, including ATF4 and its downstream products, which are related to ER homeostasis, amino acid metabolism, oxidative stress responses, and apoptosis (Rainbolt et al., 2014). Thus, ATF4 upregulates several key genes, including growth arrest and DNA damage-inducible protein 34 (GADD34) and pro-apoptotic C/EBP homologous protein (CHOP). As part of the negative feedback loop, GADD34 can dephosphorylate p-eIF2α and resume normal translation. Meanwhile, it can increase protein load during ER stress, thus aggravating stress and leading to cell death (Han et al., 2013). As the downstream product of ATF4, a large amount of CHOP is produced to trigger apoptosis after long-term ER stress (Nishitoh, 2012). Besides eIF2α, PERK can directly phosphorylate the transcription factor NRF2. Upon activation, NRF2 migrates to the nucleus, where it activates genes encoding detoxifying enzymes causing the antioxidant stress response (Cullinan and Diehl, 2004, 2006). Therefore, according to the severity and duration of ER stress, PERK activation can promote cell survival or induce cell apoptosis.

Endoplasmic reticulum stress also causes the translocation of ATF6 to the Golgi apparatus, where it is cleaved by site-1 protease (S1P) and site-2 protease (S2P). The released transcription factors activate the expression of genes related to protein folding and degradation whose products can promote protein folding, ER expansion, ERAD, and autophagy (Haze et al., 1999; Shen et al., 2002). Meanwhile, activated ATF6 can also induce the expression of XBP1 to optimize UPR (Figure 1).

### Autophagy

Different from ERAD, which is limited to degrading proteins, ER stress-induced autophagy can be divided into ER stress-mediated autophagy and ER-phagy (Song et al., 2018). The former is characterized by the degradation of damaged proteins and organelles, while the latter selectively degrades part of the ER through ATG39 and ATG40 receptors. Previous studies have shown that three branches of the UPR (IRE1α, PERK, and ATF6) modulate the formation of autophagy (Song et al., 2017, 2018). IRE1α forms a complex with TRAF2 and ASK1 to activate JNK, thus releasing Beclin-1 to drive vesicle nucleation. Meanwhile, PERK upregulates the expression of ATF4 and CHOP to induce the formation of the ATG5-ATG12-ATG16L complex, which can promote autophagy elongation. By contrast, ATF6 indirectly affects autophagy by activating XBP1 and CHOP. A feature of autophagy is the production of autophagosomes, which are degraded in lysosomes. There are three main types of autophagy in the cell: macroautophagy, microautophagy, and chaperone-mediated autophagy (Yang and Klionsky, 2010; Nakatogawa, 2020). Among these three types, macroautophagy (hereafter simply autophagy) is the most thoroughly and widely studied autophagy pathway (Cybulsky, 2017). Autophagosomes sequester and degrade damaged organelles, unfolded proteins, and aggregates; and then fuse with lysosomes for degradation of the contents (Figure 1).

Although ER stress-mediated autophagy is mainly protective, the roles of autophagy in regulating cell survival remain controversial. Moderate ER stress-mediated autophagy can reduce ER stress and promote cell survival. However, autophagy induced by excessive ER stress can aggravate cell damage and promote cell apoptosis (Denton et al., 2012; Song et al., 2017). Thus, the ultimate fate of a cell is determined by the balance between autophagy and toxic protein accumulation. On the other hand, the loss of autophagy, such as the deletion of core autophagy genes, will also change the morphology and size of the ER and induce ER stress responses (Smith and Wilkinson, 2017). In consequence, autophagy can also regulate ER function which is essential for ER homeostasis.

## Endoplasmic Reticulum Quality Control and Immune Cells

As a type of multipotent stem cell, bone marrow (BM) hematopoietic stem cells can be divided into common myeloid progenitor cells (CMPs) and common lymphoid progenitor cells (CLPs), which finally differentiate into diverse immune cells. Accumulating evidence shows that the ERQC system is essential for the development and the function of immune cells. Next, we will address the latest insights into the contribution of ERQC in the development, activation and differentiation of immune cells, although the regulation of immune response by ERQC is not limited to immune cells.

### Dendritic Cell

As the professional antigen-presenting cells, DCs process and present antigens to prime T lymphocytes for activation and proliferation. Common DC progenitors in BM can differentiate into plasmacytoid DCs (pDCs) and conventional DCs (cDCs), which include cDC1 (CD8α^+^ DCs) and cDC2 cells (Macri et al., 2018). It has been reported that XBP1 plays an important role in the development of DCs (Iwakoshi et al., 2007). In the FLT3L induction system, XBP1^–/–^ hematopoietic progenitor cells produced fewer DCs, and the apoptosis of XBP1^–/–^ DCs was increased. However, after transfection of XBP1s, DC homeostasis was restored, and development was rescued. Compared with T cells and B cells, cDCs had increased expression of IRE1α, and the transcriptional activity of XBP1 in CD8α^+^ cDCs was higher. Osorio et al. (2014) showed that the absence of XBP1 in DCs affected the structure and morphology of the ER, but its function remained unchanged (Osorio et al., 2014). Additionally, there was no significant difference in the composition and number of DC subsets in the spleen of wild-type (WT) mice and *Xbp1^*fl/fl*^Itgax^*cre*^* mice; however, the ability of CD8α^+^ cDCs to present MHC I antigens was impaired. Studies have found that the excessive activation of IRE1α in XBP1-deficient DCs led to an increase in RIDD activity but decreased the expression of Tapbp mRNA (the target of RIDD) (Osorio et al., 2014; Ramirez et al., 2019). After co-culture with OT-I T cells, the proliferation of CD8^+^ T cells was inhibited, and indicated that the cross-presentation function of CD8α^+^ cDCs was impaired. Although the expression of Tapbp is related to MHC class I antigen presentation, the specific mechanism by which RIDD affects this process remains unclear. Furthermore, it was reported that in ovarian cancer models, persistent activation of the IRE1α-XBP1 branch resulted in dysfunction of tumor-infiltrating dendritic cells (tDCs; Cubillos-Ruiz et al., 2015). Specifically, XBP1 signaling regulates the lipid metabolism and antigen presentation of tDCs, while silencing XBP1 in tDCs extends host survival by enhancing T cell anti-tumor immunity. Clearly, the IRE1α-XBP1 branch is very important for the biological functions of DC, which can further affect the activation of T cell.

Different from acute ER stress, the phosphorylation levels of eIF2α in the spleen and FLT3L BMDCs were significantly increased under steady-state conditions (Mendes et al., 2021). During the DC differentiation process, PERK was activated, which contributed to a high level of p-eIF2α. Meanwhile, cells could promote transcription through compensatory mechanisms, such as eIF2B and GADD34 (Perego et al., 2018; Kenner et al., 2019; Mendes et al., 2021). Therefore, although eIF2α negatively regulated translation, proteins were still continuously synthesized. GADD34 also coordinated with actin to regulate eIF2α phosphorylation levels, thereby affecting DC migration and cytokine secretion (Mendes et al., 2021). Furthermore, after lipopolysaccharide (LPS) stimulation, the secretion of IFN-β in PERK-deficient DCs was reduced, and the migration of DCs was impaired. However, the activation of T cells was not affected. These data indicate that although the PERK branch has no effect on DC functions and T cell activation, it is essential for type I interferon production and DC migration.

As glycoproteins on DCs, MHC class I and class II molecules bind to TCR receptors on the surface of T lymphocytes, presenting antigens to corresponding T cells (Adams and Luoma, 2013). The MHC class I heavy chain (MHC I HC) is the substrate for HRD1 degradation (Burr et al., 2011). Under normal circumstances, MHC I HC fails to bind to β2-microglobulin (β2m), which accumulates in the ER, eventually being recognized and degraded by ERAD (Hughes et al., 1997; Burr et al., 2011). Burr et al. (2013) showed that the SEL1L/HRD1/UBE2J1 complex could ubiquitinate abnormally folded MHC I HC to regulate the assembly of MHC class I molecules (Burr et al., 2013). Therefore, ERAD may be necessary for the expression of MHC class I molecules in DCs. Additionally, it was reported that B-lymphocyte-induced maturation protein 1 (BLIMP1) is the target of HRD1 ubiquitination and degradation, which can inhibit the transcription of CIITA and MHC-II genes (Yang et al., 2014). The deletion of HRD1 in DCs led to the accumulation of BLIMP1, which in turn inhibited the expression of MHC-II and CD4^+^ T cell proliferation. Interestingly, the deletion of HRD1 had no significant effect on the survival of DCs, indicating that it can specifically degrade substrates without affecting the final fate of cells. So, HRD1 appears to regulate the expression of the MHC complex through two different mechanisms. Although the specific role of ERAD in MHC-I antigen presentation remains unclear.

It is well known that DCs can process and load exogenous antigens with MHC-I molecules through cross-presentation pathways to activate naïve CD8^+^ T cells. The exogenous protein is internalized by the phagosome in DC and transported to the cytosol, where it is degraded by UPS. The generated antigen peptides are then translocated into the ER and combined with MHC-I molecules and finally loaded onto the surface of DC (Grotzke et al., 2017; Blander, 2018). Although studies have found that p97, a component of ERAD, played a role in cross-presentation retrotranslocation, HRD1 and BiP were not involved in this process (Grotzke and Cresswell, 2015). In addition, it remains unclear whether ERAD participates in the pruning and loading of peptide-MHC-I complexes (Table 1). Thus, based on these findings, determining the role of ERAD in DC cross-presentation will be an interesting future study.

**TABLE 1:** ERQC regulates the differentiation and function of immune cells.

Immune Cell		ERQC				
	
		ERAD (SEL1L-HRD1)	UPR			Autophagy
		
			IRE1α	PERK	ATF6	
**DC**		HRD1 is required for CD4^+^ T cell proliferation induced by DC (Yang et al., 2014)	Required for DC development (Iwakoshi et al., 2007). Regulate DC antigen presentation function (Osorio et al., 2014; Cubillos-Ruiz et al., 2015)	Required for DC migration (Mendes et al., 2021)	Unknown	Unknown
**Macrophage**		Unknown	Regulate TLR2 and TLR4 signaling (Martinon et al., 2010), and M1/M2 polarization (Shan et al., 2017)	Regulate M1/M2 polarization (Oh et al., 2012; Yao et al., 2016; Wang et al., 2017; Yang et al., 2019)	Regulate the release of IL-6 and TNF-α (Rao et al., 2014)	Required for the anti-inflammatory response of adiponectin (Oh et al., 2020)
**MDSC**		Unknown	Regulate PMN-MDSC suppressive activity (Tcyganov et al., 2021)	Inhibiting PERK/ATF4/CHOP attenuates immunosuppressive function (Thevenot et al., 2014; Halaby et al., 2019; Mohamed et al., 2020)	Regulate PMN-MDSC suppressive activity (Tcyganov et al., 2021)	Required for LCL521 to induce MDSCs death (Liu et al., 2016)
**B Cell**		SEL1L-HRD1 complex regulates B cell development (Ji et al., 2016; Yang Y. et al., 2018)	Regulate pro-B cell and PC differentiation (Zhang et al., 2005; Zhu et al., 2019). Required for antibodies secretion (Shaffer et al., 2004; Todd et al., 2009; Tellier et al., 2016)	UFBP1 inhibits PERK activation to promote PC development (Zhu et al., 2019)	Required for antibodies secretion (Tellier et al., 2016)	Regulate immunoglobulin synthesis (Pengo et al., 2013)
**T Cell**	**CD3^+^ T Cell**	Unknown	Required for the differentiation of DN to DP (Brunsing et al., 2008)	Regulate αβ T cell development (Solanki et al., 2016)	Unknown	Regulate T cell apoptosis of SLE patients (Lee et al., 2015)
	**CD4^+^ T Cell**	HRD1 regulates CD4^+^ T cell proliferation and Th1/Th17/Treg differentiation (Xu et al., 2016, 2018)	Regulate Th1/Th2 differentiation (Kemp et al., 2013; Brucklacher-Waldert et al., 2017)	Related to CD4^+^ T cell proliferation, Th1 differentiation and IL-4 production (Scheu et al., 2006; Yang X. et al., 2018)	Unknown	Unknown
	**CD8^+^ T Cell**	Unknown	Regulate CD8^+^ T cell differentiation (Kamimura and Bevan, 2008). Associated with T cell exhaustion in the TME (Ma et al., 2019)	Regulate the anti-tumor activity of CD8^+^ T cell (Cao et al., 2019)	Unknown	Unknown
**NK and NKT**		Unknown	Required for NK cell expansion (Dong et al., 2019). Regulate NKT1 and NKT17 cell effector functions and iNKT cell activation (Govindarajan et al., 2018, 2020)	Regulate iNKT cell activation (Bedard et al., 2019)	Unknown	Unknown

### Macrophage

Macrophages are an important part of the innate immune system, which recognize and engulf pathogens through pattern recognition receptors (PRRs) to initiate cellular immune responses. Macrophages can be simply divided into two types, monocyte-derived macrophages and resident macrophages (Verschoor et al., 2012). Upon injury or infection, circulating monocytes migrate into the tissue and transform into mature macrophages, while resident macrophages can be divided into alveolar macrophages and liver Kupffer cells (KCs), etc. On the other hand, according to their activation status, macrophages can be divided into pro-inflammatory classical activated (M1) and anti-inflammatory alternate activated (M2) macrophages. XBP1 is a positive regulator of TLR2 and TLR4 signaling responses in macrophages (Martinon et al., 2010). TLRs promote the splicing of XBP1 and the production of pro-inflammatory cytokines through TNF receptor-related factor 6 (TRAF6) and NADPH oxidase NOX2, but the expression of ER stress markers do not increase. On the contrary, the PERK branch is inhibited and the expression of CHOP is reduced, which is conducive to the survival of macrophages and the initiation of immune responses. On the other hand, as a type of adipokine, accumulating evidence indicates that adiponectin exerts anti-inflammatory properties by inducing autophagy and ER stress in macrophages (Oh et al., 2020). After inhibition of ER stress by TUDCA, the induction of autophagy was impaired, and the expression of inflammatory cytokines increased. Therefore, ER stress-mediated autophagy plays a key role in the anti-inflammatory response and cytoprotection induced by adiponectin in macrophages.

It is well known that IL-4 is a key inducer of M2 macrophages and induces the activity of transcription factors such as c-Myc in macrophages, which in turn changes their migration and polarization (Brown et al., 2012; Sica and Mantovani, 2012; Li et al., 2018). As an ER membrane protein, homocysteine-inducible ER protein with ubiquitin-like domain 1 (Herpud1) is strongly upregulated in the ER stress response, and is induced by the three branches of UPR (Yamamoto et al., 2004). Moreover, Herpud1 can interact with SEL1L, HRD1, and other components of ERAD to degrade misfolded proteins (Leitman et al., 2014). A recent study has shown that IL-4 can induce the expression of the ER stress-related protein Herpud1 in M2 macrophages (Li et al., 2018). Inhibition of ER stress by 4-PBA or knockdown of the expression of Herpud1 suppressed the polarization and migration of M2 macrophages induced by IL-4. Clearly, Herpud1 plays an important role in IL-4 activated M2 polarization, but the potential mechanism remains undetermined.

As highly plastic and diverse cells, the polarized phenotype of macrophages can be transformed into each other under different microenvironments. In obesity, palmitic acid (PA) and LPS in the blood activate adipose tissue macrophages (ATMs) IRE1α branch, and then inhibit M2 and enhance M1 polarization, which ultimately lead to inflammation (Shan et al., 2017). However, the loss of IRE1α can reverse the imbalance of M1 and M2 polarization in ATMs and improve metabolism. Similarly in fatty liver, ER stress leads to the activation of the PERK branch in KCs, which induces the transformation of KCs into pro-inflammatory M1 (Yang et al., 2019). However, the absence of PERK can inhibit LPS induction of STAT1 activation and enhance IL-4 induction of STAT6 activation, thereby dampening the polarization of M1 and promoting the polarization of M2. In contrast, in patients with pulmonary fibrosis, diabetes, and allergic airway inflammation, the infiltration of M2 macrophages was increased, and the expression of ER stress markers, especially CHOP, was significantly upregulated (Oh et al., 2012; Yao et al., 2016; Wang et al., 2017). However, the loss of CHOP can inhibit the activation of STAT6 and then attenuate M2 polarization, thereby alleviating the inflammatory response. Furthermore, ATF6 activation was shown to amplify the pro-inflammatory response of TLR4 (Rao et al., 2014). Specifically, ATF6-mediated ER stress enhances the pro-inflammatory property of TLR4 by enhancing NF-κB signaling, while inhibiting ATF6 can alleviate ER stress and decrease the production of IL-6 and TNF-α. This indicates that there is a regulatory mechanism between ATF6 and TLR4 to prevent excessive inflammation.

Collectively, these findings indicate that the ER stress branches can regulate the polarization of M1 and M2, which can affect the progression of a variety of chronic diseases (Table 1). Further works are needed to explore the specific effect of macrophage ER stress in different diseases, which is expected to provide information for clinical targeted therapy.

### Myeloid-Derived-Suppressor Cell

Myeloid-derived-suppressor cells are immature myeloid cells with immune suppressive activity, which can be divided into polymorphonuclear MDSC (PMN-MDSC) and monocytic MDSC (M-MDSC), and the large accumulation of MDSCs in cancer patients is the main factor limiting the efficacy of immunotherapy. Recently, apoptosis pathway was shown to play a key role in MDSCs homeostasis. Condamine et al. (2014) found that MDSCs in tumor-bearing mice had increased apoptosis and poor survival, which was associated with the increased expression of TRAIL receptors (TRAIL-Rs; Condamine et al., 2014). Subsequent further study showed that MDSCs from cancer patients exhibited higher ER stress levels, and the expression of TRAIL-Rs was upregulated, while targeting TRAIL-Rs improved the anti-tumor effect. In contrast, the acid ceramidase inhibitor LCL521 induced MDSCs death through an apoptosis-independent mechanism death (Liu et al., 2016). Specifically, LCL521 leads to the abnormal accumulation of autophagic vesicles and ER stress, which impaired autophagic flux and induced MDSCs death.

In addition, the immune function of MDSCs is also significantly affected by ER stress. The induction of ROS was impaired in CHOP-deficient MDSCs, which not only inhibited the immunosuppressive function, but also initiated T cell response to induce anti-tumor immunity (Thevenot et al., 2014). This transition was mediated through C/EBPβ and p-STAT-3 signaling. Although CHOP induction is mainly driven by ATF4, it is unclear whether ATF4 regulates MDSC function. A recent study showed that the GCN2-ATF4 axis drives the polarization of tumor-associated macrophages and the immunosuppressive functions of tumor-infiltrating MDSCs that attenuate anti-tumor immunity (Halaby et al., 2019). Likewise, Mohamed et al. (2020) found that in PERK-deficient tumor MDSCs, reduced NRF2 signaling elicited the accumulation of mtDNA and subsequently stimulated the STING pathway, which transformed MDSCs into cells that promote CD8^+^ T cell responses.

Of note, IRE1α-XBP1 and ATF6 also play an important role in altering the function of MDSCs. PMN-MDSCs in cancer patients exhibited higher expression of lectin-type oxidized LDL receptor-1 (LOX-1) and ER stress-related genes than neutrophils (PMNs) (Condamine et al., 2016). XBP1 inhibitor B-109 can suppress the upregulation of LOX-1 and the suppressive activity in PMN induced by ER stress. The function of PMN-MDSCs appears to be affected by IRE1α-XBP1. Supporting this observation, Tcyganov et al. (2021) found that IRE1α and ATF6 can regulate the suppressive activity of PMN-MDSC in tumors. The deletion of IRE1α and ATF6 abrogated PMN-MDSC suppressive activity and delayed tumor progression. Instead, the function of M-MDSC was controlled by IFN-γ signaling and was dispensable for ER stress. These findings indicate that UPR promotes immune evasion by regulating tumor MDSCs survival and immunosuppressive functions, which is related to cancer progression (Table 1).

### B Cell

During the differentiation of B lymphocytes, pro-B cells differentiate into large pre-B cells expressing the pre-B cell receptor (pre-BCR). After several divisions, large pre-B cells differentiate into small resting pre-B cells and finally develop into immature B cells expressing BCR (Qi et al., 2017). Recent studies have shown that the SEL1L-HRD1 ERAD complex terminated pre-BCR signaling in a BiP-dependent way and indicated that ERAD regulated the development of B cells (Ji et al., 2016; Yang Y. et al., 2018). As an important node for B cell development, pre-BCR consists of IgH and the surrogate light chain (SLC), which contains λ5 and VpreB (Zhang et al., 2004). The silent expression of SLC in large pre-B cells could downregulate pre-BCR signaling and differentiate large pre-B cells into small pre-B cells. In the deletion of SEL1L-HRD1, the pre-BCR signaling complex was accumulated, leading to the strengthened proliferation of large pre-B cells and developmental defects in B cells. Eventually, the number of small pre-B cells decreased (Ji et al., 2016; Yang Y. et al., 2018). Unfortunately, it is unclear how ERAD differentiates between the pre-BCR and BCR complex expression on B cells.

Plasma cells (PCs) are terminally differentiated B lymphocytes responsible for secreting antibodies, which are essential components of humoral immunity. To synthesize large amounts of immunoglobulins, new PCs must increase the size of the ER and potentially induce ER stress. Thus, the UPR, as a complex signal transduction pathway might play an important role in this process. Indeed, the lack of XBP1 in B cells severely impaired the synthesis and secretion of antibodies, but was dispensable for the development of PC and memory B cells (Todd et al., 2009). In addition, as an essential transcription factor for PC differentiation, BLIMP1 promoted the splicing of XBP1 by regulating ATF6 and IRE1α, which was required for antibody secretion (Shaffer et al., 2004; Tellier et al., 2016). However, beyond affecting the differentiation of PC, IRE1α can also regulate the differentiation of pro-B cells by promoting Ig genes rearrangements and the formation of BCRs (Zhang et al., 2005). Beyond the role of UPR in PCs, it is now recognized that autophagy is also essential for the differentiation of PCs (Sandoval et al., 2018). In a mouse model lacking the autophagy gene ATG5, PCs were shown to have an expanded ER and stronger ER stress (Pengo et al., 2013). Despite enhanced immunoglobulin synthesis, the antibody titers and survival of PCs decreased, indicating that autophagy is essential for PC homeostasis, which can limit immunoglobulin synthesis and optimize humoral immunity (Table 1).

As reported in most literature, the PERK pathway is not required for antibody secretion. Zhu et al. (2019) showed that UFBP1 can suppress the PERK pathway and promote the development of PCs (Zhu et al., 2019). Compared with WT B cells, the expression of ATF4 was significantly increased in UFBP1-deficient B cells (Zhu et al., 2019). Meanwhile, PC frequencies in the spleen and BM were comparable between WT, *Perk^*F/F*^CD19^*cre*^*, and *Perk^*F/F*^Ufbp1^*F/F*^CD19^*cre*^* mice, but obviously lower in *Ufbp1^*F/F*^CD19^*cre*^* mice. This result confirmed the previous conclusion that the absence of PERK does not affect the differentiation of PCs. Moreover, the IRE1α-XBP1 branch was shown to regulate the expression of UFBP1 to promote ER expansion (Zhu et al., 2019). These findings indicate that UFBP1 affects the development and function of PCs by participating in different branches of the UPR pathway. However, the specific mechanism for UFBP1 regulating PERK remains unidentified. UFBP1 may inhibit PERK activity through a pathway unrelated to ER stress, thereby promoting the differentiation of naïve B cells into PCs.

Orosomucoid-like 3 (ORMDL3) is a new family of ER proteins, which was reported to play an important role in maintaining ER homeostasis by regulating the UPR response (Dang et al., 2017). Compared with WT mice, the level of B-cell activating factor (*Baff*) mRNA in spleen cells and serum in *Ormdl3*^–^*^/^*^–^ mice were significantly decreased, which was crucial for B cell development and function (Parameswaran et al., 2010; Dang et al., 2017). The loss of *Baff* influenced the differentiation of T1 B cells to T2 B cells, thereby impairing B cell development, and ultimately decreasing the number of mature B cells (Dang et al., 2017). Beclin-1 is a core component of the class III PI3K complex and is essential for the initiation of autophagy. Experiments showed that ORMDL3 could induce autophagy and inhibit apoptosis through Beclin-1 to promote cell survival. In addition, Dang et al. (2017) found that ORMDL3 mediated ER stress mainly through the ATF6 pathway (Dang et al., 2017). Compared with HEK293 cells overexpressing ORMDL3, siRNA-mediated silencing of ATF6 decreased the expression of Beclin-1 and LC3-II. Collectively, ORMDL3 promotes autophagy and inhibits apoptosis through the ATF6-Beclin-1 autophagy regulatory pathway, and ORMDL3 may be important for B cell differentiation and maturation.

Of note, through the analysis of activated B cells, Gaudette et al. (2020) found that B cells upregulated many UPR-related genes before secreting robust antibodies (Gaudette et al., 2020). Instead of XBP1, mTORC1 drives the expression of these genes. Before the XBP1 arm is completely induced, activated B cells initiate the mTORC1 signal, independent of XBP1, upregulating many UPR components to lay the foundation for the early activation of UPR. For instance, by early upregulation of BiP expression, the ER function could be enhanced, reducing the early PCs ER stress caused by the synthesis of large amounts of Ig (Gaudette et al., 2020). This hypothesis reveals aspects that are overlooked in the early PC differentiation process. These results provide suggestions for the identification of early PCs and mature PCs.

Consistent with its critical role in normal PC biology, UPR aberrant activation is extensively related to multiple myeloma (MM). Studies have shown that overexpression of XBP1 appears to promote the initiation of MM. Compared with control mice, *Eμ-XBP1s* mice exhibited enhanced B cell proliferation and aberrant transformation of PCs (Carrasco et al., 2007). Transcriptome analysis showed that the expression of genes related to the pathogenesis of MM was generally upregulated, indicating the driving role of XBP in MM. Similarly, Patterson et al. (2008) found that the activities of XBP1 and ATF6 were abnormally increased in MM cells, but the PERK pathway inducing apoptosis was not activated. The role of PERK in MM appears to be unclear. Only recently has PERK been proven to play an important role in MM growth. The high expression of BLIMP1 in MM cells inhibited the activation of IRE1α-ASK1 and PERK pathways, therefore, MM cells can survive for a long term without apoptosis (Lin et al., 2012; Liu et al., 2020). These findings indicate that UPR appears to regulate MM differently. Based on these, many small molecule inhibitors or proteasome inhibitors (PIs) have been studied in MM. For example, as a potent PI, bortezomib triggers ER stress by blocking ERAD, and then upregulates CHOP to induce apoptosis (Obeng et al., 2006). However, attributed to the existence of PI resistance, MM still remains incurable. The reason for the failure of PI treatment appears to be related to IRE1α. Leung-Hagesteijn et al. (2013) found that the lack of IRE1α-XBP1 signaling blocked the maturation of PCs, which can promote PI resistance. Therefore, although IRE1α kinase inhibitors can reduce the viability of MM cells, its role in the treatment of MM is controversial.

On the other hand, as a pro-survival mechanism, autophagy promotes MM cells proliferation and survival by degrading protein aggregates (Milan et al., 2016). Based on this, the current researches of autophagy in anti-myeloma treatment mainly focus on two methods (Yun et al., 2017). The first is to induce persistent ER stress and trigger apoptosis by inhibiting autophagy or other protein degradation pathways. The second is to induce cell death by enhancing autophagy. Thus, based on the importance of autophagy in MM cells survival, targeting autophagy may become a new approach for MM treatment.

### T Cell

In the thymus, immature T cells gradually differentiate into CD4^–^CD8^–^ double negative (DN) cells, CD4^+^CD8^+^ double positive (DP) cells, and finally develop into CD4^+^, or CD8^+^ single positive (SP) cells with MHC restricted recognition and autoantigen tolerance (Brunsing et al., 2008). During these developmental stages, cells transition from quiescence to proliferation, accompanied with increased protein synthesis, which may trigger ER stress. Studies have shown that IRE1α was active in the DP stage, and the loss of IRE1α in BM pluripotent stem cells prevented the differentiation of DN to DP (Brunsing et al., 2008). Moreover, ribosomal protein L22 (RPL22) was shown to promote the development of αβ T cells by inhibiting ER stress responses (Anderson et al., 2007; Solanki et al., 2016; Kemp and Poe, 2019). The deletion of RPL22 aggravated ER stress in αβ T cells, leading to developmental arrest through the selective induction of p53 (Solanki et al., 2016). Downregulation of the PERK signaling pathway could inactivate p53 induction, thereby rescuing the developmental defects of αβ T cells. These results indicate that PERK appears to affect the development of αβ T cells by regulating the expression of p53, although the mechanism for the induction of p53 remains unclear. On the other hand, the activation of TCR induced ER stress, resulting in the upregulation of the expression of ER chaperones Gp96 and BiP (Thaxton et al., 2017). The lack of Gp96 hindered the differentiation of CD4^+^ T cells. While Gp96 is the target molecular chaperone of ATF6, it remains unknown whether ATF6 is involved in the differentiation of CD4^+^ T cells.

It is well known that disrupted T cell homeostasis is one of the pathogenic mechanisms of systemic lupus erythematosus (SLE). ER stress induced by ultraviolet irradiation or viral infection is responsible for SLE flare-ups (Hirabayashi et al., 2007). A previous study found that after TG induced ER stress, compared with healthy people, the expression of ER stress markers and autophagy decreased in T cells of SLE patients, while the expression of CHOP increased (Lee et al., 2015). Eventually the level of T cell apoptosis was higher and homeostasis was dysregulated. However, the effect of ER stress-induced autophagy and apoptosis on T cell function and subsets remains unclear, and further research is needed to provide more effective information for the clinical treatment of SLE (Table 1). Beyond affecting T cell development and survival, ERQC is also closely related to the differentiation and effector functions of T cells.

#### CD4^+^ T Cell

CD4^+^ T cells are at the center of the adaptive immune response which activation is strictly regulated. When stimulated with antigen, naïve CD4^+^ T cells are induced into activation, proliferation, and cytokine secretion and further differentiate into different subsets, including T helper type 1 (Th1), Th2, Th17, and regulatory T cells (Tregs). HRD1 was shown to regulate the development and differentiation of CD4^+^ T cells (Xu et al., 2016). Loss of HRD1 in T cells results in the accumulation of p27^*kip1*^ protein, which arrests the cell cycle at G1/G0, and then disrupts T cells development and inhibits the proliferation of CD4^+^ T cells (Xu et al., 2016). However, the deletion of p27^*kip1*^ in HRD1-deficient T cells does not rescue IL-2 production and Th1 or Th17 differentiation. Considering that E3 ubiquitin ligase usually targets a variety of substrates to achieve its physiological functions, HRD1 may modulate other substrates to regulate CD4^+^ T cell differentiation.

On the other hand, it has been confirmed that ATF4 plays a role in Th1 differentiation. During the activation of T cells, the increased level of glutathione promotes the expression of ATF4, thereby enhancing protein synthesis to ensure the demand for T cell proliferation (Yang X. et al., 2018). Compared with WT mice, the level of total glutathione in *Atf4*^–^*^/^*^–^ mice was significantly reduced, resulting in impaired redox homeostasis and reduced CD4^+^ T cell proliferation (Yang X. et al., 2018). Also, under Th1 polarization conditions, the secretion of IFN-γ was reduced. But, IL-17 secretion remained unchanged under Th17 polarization conditions. In the experimental allergic encephalomyelitis (EAE) model, the Th1 response in *Atf4*^–^*^/^*^–^ mice was impaired, while Th17 levels were increased, suggesting that ATF4 is necessary for Th1 differentiation. In addition, Brucklacher-Waldert et al. (2017) confirmed that XBP1 can affect Th17 differentiation efficiency. Mice lacking XBP1 showed impaired Th17 differentiation and slower EAE progression. Collectively, these findings indicate that ERQC can regulate the differentiation of Th1 and Th17, which may provide new insights for the development of autoimmune diseases.

Moreover, the IRE1α-XBP1 branch is also involved in Th2 differentiation. Through transcriptome analysis, it has been found that the expression of XBP1 is significantly upregulated when CD4^+^ T cells are activated and differentiate into Th2 (Kemp et al., 2013; Pramanik et al., 2018). After treating CD4^+^ T cells with IRE1α RNase inhibitor 4μ8c, the levels of IL-4, IL-5, and IL-13 decreased under Th2 polarization. Interestingly, the PERK-eIF2α branch appears to be related to the secretion of IL-4. TCR stimulation resulted in increased phosphorylation of eIF2α in Th2 cells, but IL-4 protein expression was not detected (Scheu et al., 2006). Unexpectedly, restimulation of Th2 cells showed increased dephosphorylation of eIF2α, and the translation block was relieved, which promoted the production of IL-4. This indicates that the promotion or inhibition of ER stress affect IL-4 secretion, and UPR appears to regulate Th2 effector function in different ways.

Expression of the transcription factor Foxp3 controls the differentiation and function of Tregs. Under inflammatory conditions, ER stress downregulates Foxp3 expression. Eventually, Tregs fail to maintain stable expression, and their function is weakened (Rudensky, 2005; Xu and Fang, 2020). A recent study reported that IRE1α expression was increased in *Hrd1^*fl/fl*^-Foxp3^*cre*^* mice, and Foxp3 expression was impaired. After treatment with IRE1α inhibitor, the expression of Foxp3 was rescued, and the function of Tregs was restored (Xu et al., 2019). Therefore, inhibiting ER stress responses mediated by IRE1α or HRD1 might maintain Tregs homeostasis (Table 1).

#### CD8^+^ T Cell

CD8^+^ cytotoxic T cells are the main effector cells of anti-tumor immunity. Adverse conditions in the tumor microenvironment (TME), such as nutrient deprivation, hypoxia, low pH, and ROS, disrupt protein folding, and aggravate ER stress (Urra et al., 2016; Oakes, 2020). Studies have shown that the splicing of XBP1 was increased in CD8^+^ T cells during acute infection, which can promote the expression of KLRG1 and enhance effector function (Kamimura and Bevan, 2008). However, the loss of XBP1 led to a decrease in the number of KLRG1^*high*^ effector cells, and the terminal differentiation of CD8^+^ T cells was hindered. In contrast, cholesterol in tumor tissues induced the expression of XBP1 in tumor-infiltrating CD8^+^ T cells (Ma et al., 2019). Subsequently, XBP1 induced T cell exhaustion by upregulating the expression of a variety of inhibitory receptors (such as PD-1, 2B4). Therefore, although XBP1 can promote the differentiation of CD8^+^ cytotoxic T cells, prolonged ER stress is harmful to T cells in the TME.

Contrary to the role of UPR in the development and differentiation of T cells, CHOP is responsible for the impaired tumor immunity. In CD8^+^ T lymphocytes of patients with advanced ovarian cancer, CHOP expression was elevated, and associated with its poor clinical response (Cao et al., 2019). The accumulation of ROS in the TME could activate PERK and induce the expression of ATF4, which resulted in the upregulation of CHOP. A further study found that the pro-apoptotic factor CHOP can bind and inhibit the transcriptional activity of Tbx21, thereby downregulating the expression of T-bet protein, leading to a decrease in the cellular activity of effector CD8^+^ T cells (Cao et al., 2019). CD8^+^ T cells lacking CHOP showed enhanced anti-tumor activity and a delayed tumor growth. Although the loss of CHOP weakens the immunosuppressive function of tumor MDSCs and enhances the activity of tumor infiltrating T cells, ER stress can also modulate tumor cell survival. Therefore, the efficacy of the clinical application of CHOP inhibition needs to be further tested.

On the other hand, in viral infection condition, naïve CD8^+^ T cells recognize the specific antigen presented by DCs, and then activate and divide into effector T cells. After the pathogen is cleared *in vivo*, most effector CD8^+^ T cells die, and only a few differentiate into memory T cells (Puleston et al., 2014). During the transition from effector to memory cells, excess proteins accumulate in cytosol, and ER load dramatically increases. Studies have found that autophagy activity increases during this contraction phase, which affects the development of memory CD8^+^ T cells through lipid metabolism (Xu et al., 2014). Given that autophagy modulates ER homeostasis of T lymphocytes, it may also regulate the differentiation of memory CD8^+^ T cells through ER stress; a point which deserves further study (Table 1).

### Natural Killer and Natural Killer T Cell

Natural killer cells are special innate lymphocytes derived from CLPs, which have cytotoxic functions similar to cytotoxic T lymphocytes (CTLs), but lack TCR receptors. To date, NK cells play an indispensable role in viral infection and anti-tumor immunity. It is reported that mouse cytomegalovirus (MCMV) infection activates IRE1α-XBP1 in NK cells and drives cell proliferation through the c-Myc signaling pathway (Dong et al., 2019). Although the loss of IRE1α has no effect on NK cell activation and apoptosis, the proliferation and expansion of these cells are impaired. Moreover, XBP1 can promote the survival and enhance the cytotoxic activity of IL-15-driven NK cells (Wang et al., 2019). This indicates that ER stress is an important factor in the expansion of NK cells and suggests that IRE1α-XBP1 may be a potential target for improving the therapeutic effect of expanded NK cells *in vitro* (Table 1).

Natural killer T (NKT) cells are a special type of T lymphocyte that express TCR and the NK cell receptors. Present studies divide NKT cells into two subsets, most of which are type I NKT cells, also known as invariant NKT (iNKT) cells (Kumar and Delovitch, 2014). Unlike conventional T cells, NKT cells are activated by the lipid antigen presented by CD1d and release cytokines rapidly (Wu and Van Kaer, 2011). Current data indicate that iNKT cells can differentiate into NKT1, NKT2, and NKT17, which play different roles in immunity (Hogquist and Georgiev, 2020). Govindarajan et al. (2018) found that TCR-dependent stimulation of iNKT sublineages resulted in the increased expression of XBP1 in NKT1 and NKT17 (Govindarajan et al., 2018). Although IRE1α had no effect on the development and maturation of resting iNKT cells, the deletion of IRE1α could inhibit cytokine production of NKT1 and NKT17 subsets, which suggested that IRE1α can modulate NKT1 and NKT17 cell effector functions. In contrast to the pro-inflammatory effect of PA in macrophages, a recent study found that PA enhanced the degradation of RIDD on *T-bet* and *gata-3* mRNA by inducing ER stress in iNKT cells, thereby suppressing the production of IL-4 and IFN-γ and attenuating arthritis (Ko et al., 2017). In addition, ER stress induced IRE1α and PERK-dependent branches can increase lipid antigens bound to CD1d molecules, thereby enhancing iNKT cell activation (Bedard et al., 2019; Govindarajan et al., 2020). This indicates that ER-stressed APCs can affect iNKT cell activation via CD1d. Given that self-lipids participate in the positive selection of thymic iNKT cells, it is possible that ER stress in thymocytes may impact self-lipids presented by CD1d to further affect the positive selection and development of iNKT cells (Table 1).

## Pharmacological Regulation of Endoplasmic Reticulum Stress

Drugs that regulate ER stress have been reviewed in detail elsewhere (Hetz et al., 2019; Chen and Cubillos-Ruiz, 2021). Therefore, this section will briefly introduce these compounds. The acute activation of the IRE1α branch under ER stress has a protective effect, but its chronic activation is related to diseases such as cancer. There are two kinds of IRE1α inhibitors, RNase inhibitor and kinase inhibitor (Hetz et al., 2019). RNase inhibitors include 4μ8c, MKC3946, and STF-083010, which can block the splicing of XBP1, and the kinase inhibitors include KIRA6 and AMG18. Studies have shown that the use of IRE1α inhibitors may become a potential treatment strategy. For example, STF-083010 and AMG18 inhibit the proliferation of tumor cells in MM and pancreatic neuroendocrine tumors, respectively (Papandreou et al., 2011; Moore et al., 2019). In addition, B-I09 (4μ8C analogs) can induce apoptosis of B-cell leukemia cells (Tang et al., 2014). However, there are also reports showing that 4μ8c exhibits off-target effects (Sato et al., 2017). 4μ8c treatment of pancreatic β-cells blocked the secretion of insulin, indicating that 4μ8c has other side effects unrelated to the inhibition of IRE1α activity.

Unlike GSK2606414 and GSK2656157, AMG44 and AMG52 are highly selective PERK inhibitors without pancreatic toxicity (Smith et al., 2015). PERK kinase inhibitors are widely used in a variety of diseases, which can suppress the growth of human tumor xenografts and improve the progression of neurodegenerative diseases including frontotemporal dementia (Atkins et al., 2013; Radford et al., 2015). However, these compounds have a dose-dependent defect and further clinical studies are needed to evaluate their potential side effects. Apart from directly inhibiting PERK signaling, ISRIB is a potent eIF2α inhibitor, which significantly inhibits the progression of prostate cancer by blocking the phosphorylation signaling of eIF2α (Nguyen et al., 2018). Notably, activation of PERK and phosphorylation of eIF2α can also play a protective role. CCT020312 and its derivative MK-28 activated the PERK pathway and improved the symptoms of Huntington’s disease in mice (Ganz et al., 2020). However, the activation mechanism remains undefined and needs further exploration. Moreover, similar to Salubrinal in preventing eIF2α dephosphorylation, guanabenz and its analog Sephin1 target GADD34 to delay translation recovery and relieve amyotrophic lateral sclerosis (Das et al., 2015). Paradoxically, studies showed that guanabenz and Sephin1 do not inhibit GADD34 activity, and it is necessary to further explain how these compounds induce sustained eIF2α phosphorylation (Crespillo-Casado et al., 2017, 2018). Although the PERK activators have been shown to have potential therapeutic value, prolonged phosphorylation of eIF2α may induce the expression of pro-apoptotic genes. Therefore, in order to obtain the desired therapeutic effect, these factors must be carefully considered.

Contrary to the role of IRE1α and PERK in diseases, ATF6 appears to have less effect on organism functions. Recently, with the development of ATF6 compounds, the unique therapeutic potential has gradually emerged. Ceapin-A7 is a highly effective ATF6 inhibitor, which prevents the movement of ATF6 by inducing the accumulation of ATF6 in the ER, but the exact target is still unclear (Gallagher et al., 2016). Alternatively, compounds 147 and 263 have been shown to specifically activate ATF6, of which 147 can protect the heart from ischemia/reperfusion injury (Blackwood et al., 2019).

Thus, although regulators of the three UPR branches have been developed, each method faces the problem of targeted toxicity and mechanism of action. In the future, it is necessary to define the role of UPR signaling in different physiological and pathological conditions and optimize the dosing regimen to promote the clinical application of UPR modulators.

## Conclusion and Perspective

Protein homeostasis is essential for the normal survival of immune cells. However, the accumulation of misfolded proteins above a threshold will induce ER stress. To restore homeostasis, cells reduce protein synthesis through ERQC, including activating the UPR response, ERAD and autophagy. Although an increasing number of data recognize that ERQC plays an important role in the pathogenesis of tumors and inflammatory disease, its exact role in the biology of specific immune cells still waits for further exploration.

Moreover, the application of small molecule inhibitors has confirmed that UPR is involved in various diseases, there are still many problems to be solved. For example, UPR targeted therapy will have off-target effects, and the UPR in a state of over-activation or inhibition can also cause harmful effects. In the future, it may be possible to adopt short-term treatment or local delivery strategies to reduce organ toxicity.

Taken together, regulation of the ERQC system may constitute a new method for promoting immunity, but more investigation is needed.

## Author Contributions

SX conceived the idea and designed the work. YJ designed and drafted the manuscript. ZT perfected the literature search and edited the manuscript. HC helped in the concept discussion. All authors contributed to the manuscript revision and approved the submitted version.

## Conflict of Interest

The authors declare that the research was conducted in the absence of any commercial or financial relationships that could be construed as a potential conflict of interest.

## Publisher’s Note

All claims expressed in this article are solely those of the authors and do not necessarily represent those of their affiliated organizations, or those of the publisher, the editors and the reviewers. Any product that may be evaluated in this article, or claim that may be made by its manufacturer, is not guaranteed or endorsed by the publisher.

## Abbreviations

ERQC: Endoplasmic reticulum quality control
ERAD: Endoplasmic reticulum-associated degradation
UPR: Unfolded protein response
DCs: Dendritic cells
MDSCs: Myeloid-derived-suppressor cells
NK: Natural killer
ER: Endoplasmic reticulum
IRE1α: Inositol-requiring enzyme 1α
PERK: Protein kinase RNA-like ER kinase
ATF6: Activating transcription factor 6
SER: Smooth endoplasmic reticulum
RER: Rough endoplasmic reticulum
UPS: Ubiquitin-proteasome system
ALS: Autophagic-lysosomal system
GRP78/Bip: Glucose-regulated protein 78
XBP1: X-box-binding protein 1
TRAF2: Tumor-necrosis factor-α-receptor-associated factor 2
ASK1: Apoptosis signal-regulating kinase 1
JNK: JUN N-terminal kinase
RIDD: Regulated IRE1-dependent decay
eIF2α: Eukaryotic initiation factor 2α
GADD34: Growth arrest and DNA damage-inducible protein 34
CHOP: C/EBP homologous protein
BM: Bone marrow
CMPs: Common myeloid progenitor cells
CLPs: Common lymphoid progenitor cells
BLIMP1: B-lymphocyte-induced maturation protein 1
KCs: Kupffer cells
PA: Palmitic acid
ATMs: Adipose tissue macrophages
PMN-MDSC: Polymorphonuclear MDSC
M-MDSC: Monocytic MDSC
TRAIL-Rs: TRAIL receptors
LOX-1: Lectin-type oxidized LDL receptor-1
Pre-BCR: Pre-B cell receptor
PCs: Plasma cells
UFBP1: UFM1 binding protein 1
ORMDL3: Orosomucoid-like 3
Baff: B-cell activating factor
MM: Multiple myeloma
PIs: Proteasome inhibitors
RPL22: Ribosomal protein L22
SLE: Systemic lupus erythematosus
ROS: Reactive oxygen species
EAE: Experimental allergic encephalomyelitis
TME: The tumor microenvironment
MCMV: Mouse cytomegalovirus
iNKT: Invariant NKT.
